# Optimized Extract from* Corylopsis coreana* Uyeki (Hamamelidaceae) Flos Inhibits Osteoclast Differentiation

**DOI:** 10.1155/2018/6302748

**Published:** 2018-03-06

**Authors:** Yongjin Lee, Jung-Eun Kim, Kwang-Jin Kim, Seung-Sik Cho, Young-Jin Son

**Affiliations:** ^1^Department of Pharmacy, Sunchon National University, Jeonnam, Suncheon 57922, Republic of Korea; ^2^Department of Pharmacy, College of Pharmacy, Mokpo National University, Muan, Jeonnam 58554, Republic of Korea

## Abstract

Osteoporosis is a metabolic disorder that decreases the stability against fractures of the spine, femur, and radius by weakening the strength and integrity of bones. Receptor activator of nuclear factor-kappa B ligand signaling ultimately activated nuclear factor-activated T cells c1, a major transcription factor for osteoclast formation. This study researched the effects of* Corylopsis coreana (C. coreana) *Uyeki flos extracts on the antiosteoclastic potential of macrophages and the phytochemicals contained therein. The alcoholic extract of* C. coreana *Uyeki flos inhibited the differentiation of osteoclast. We carried out the experiments of the pattern of differentiation of osteoclasts based on the alcoholic percentage of extracts. Among them, 80% alcoholic extract showed the highest inhibitory effect. The alcoholic extract was composed of phytochemicals such as bergenin, quercetin, and quercitrin. This extract inhibited not only mRNA expression levels of NFATc1, osteoclast-associated receptor (OSCAR), cathepsin K, and tartrate-resistant acid phosphatase (TRAP), but also the translational expression of NFATc1. The inhibitory effect for osteoclast differentiation of the alcoholic extract was confirmed using the resorption pit assay. This is the first scientific report of the antiosteoclastic effects of* C. coreana* Uyeki flos extract, which can be applied therapeutically for the treatment of osteoporosis.

## 1. Introduction

Osteoporosis is a metabolic disease in which the bone integrity and strength are weak due to qualitative changes in the bone microstructure, which decreases the stability against fractures of the spine, femur, and radius. In diseases, such as osteoporosis, the number of osteoclasts is increased resulting in a decrease in bone density. Moreover, in inflammatory conditions, for example, rheumatoid arthritis and periodontitis, osteoclast formation is activated with concomitant bone destruction. The differentiation of osteoclasts is regulated by receptor activator of nuclear factor-kappa B ligand (RANKL), a cytokine essential for the bone metabolism [1]. The interaction between RANK and RANKL activates the continuity of intracellular molecular phenomena directly, leading to osteoclastogenesis. The osteoclast differentiation pathway by RANKL includes NF-*κ*B, mitogen-activated protein kinase (MAPK), including c-Jun N-terminal kinase (JNK), extracellular signal-regulated kinase (ERK), and p38 [2]. This signal transduction can interact with nuclear factor-activated T cells c1 (NFATc1), a master regulator of osteoclastogenesis, leading to translocation into the nucleus [3]. NFATc1, a member of the transcription factors NFAT family, is increased significantly during RANKL-induced osteoclastogenesis [4].

*C. coreana *Uyeki flos belongs to the Hamamelidaceae (witch hazel) family and is cultured as a medicinal plant in China and South Korea [5].* C. coreana *Uyeki flos has been utilized as a traditional medicine to treat chills, nausea, dizziness, and heart pounding [5, 6] but there are few scientific reports of* C. coreana* Uyeki flos. In a previous study, some compounds from the leaves of* C. coreana *Uyeki flos were extracted and identified. It was discovered that they had antioxidative and antiproliferative components [5]. Recently, the authors identified flavonoids and isocoumarin in the flower extract of* C. coreana *Uyeki flos and reported their antimicrobial, antioxidant, and antihyperuricemic activity [7, 8]. On the other hand, to the best of the authors' knowledge, there is a paucity of reports on the antiosteoclastic activity of* C. coreana* Uyeki flos.

Therefore, this study examined the osteoclastic activity of the alcoholic extracts of* C. coreana *Uyeki flos and identified the active constituents responsible for the antiosteoclastic effects using the relevant* in vitro* systems. This study could provide a basic information for the complementary and alternative herbal medicines for the treatment of osteoporosis and its related diseases.

## 2. Materials and Methods

### 2.1. Plant Materials

Samples of* C. coreana *Uyeki flos were collected in May 2013 near Jogye Mountain, in Jeonnam Province, Korea. A voucher specimen (MNUCSS-CC-01) was deposited in Mokpo National University (Muan, Republic of Korea). The air-dried and powdered samples of* C. coreana* Uyeki flos (10 g) were extracted twice with diverse percentages of ethanol (100 mL) at RT for 3 days. After filtration, the resulting ethanol solution was evaporated, freeze-dried, and stored at −50°C. The crude extract was resuspended in ethanol and filtered using a 0.4 *μ*m membrane filter. All samples were used for the optimization of extract and* in vitro* experiments.

### 2.2. Constituent Purification and Profiling by High-Performance Liquid Chromatography (HPLC)

The air-dried, powdered extract of* C. coreana* Uyeki flos was sonicated for 3 hrs with ethanol. After filtration, the ethanol was evaporated and suspended in distilled water and then defatted with an* n*-hexane, ratio of 1 : 1 (v/v). The aqueous layer was partitioned with ethyl acetate (2 × 200 mL). The evaporation residue (2 g) was subjected to flash silica gel chromatography, using an* n*-hexane : EtOAc solvent system (9 : 1~3 : 1), to afford 10 fractions. Fractions 2, 3, and 4 were subjected to further RP-C18 silica gel chromatography, using an acetonitrile : water (10 : 100) eluent system, to afford bergenin (BER), quercitrin (QCIT), and quercetin (QCT). The compounds were further purified by preparative thin layer column chromatography for analytically acceptable purity. Constituent-profiling of the* C. coreana* Uyeki flos samples by HPLC analysis was performed as previously described [8]. All analysis was conducted using an Alliance 2695 HPLC system (Waters; Millford, MA, USA) equipped with a photodiode array detector. The analytical column used was an Agilent Zorbax extended C18 column (5 *μ*m, 150 mm × 5 mm) with a mobile phase consisting of solvents A (acetonitrile) and B (water containing 0.2% phosphoric acid) and employing gradient elution (from 10/90 to 100/0, v/v) at a flow rate of 0.8 mL/min. The column temperature was maintained at 25°C, and the detection wavelength was set to 350 nm for BER, QCT, and QCIT. The mobile phase was filtered through a 0.22 *μ*m filter and degassed.

### 2.3. Cell Cultures and Osteoclast Differentiation

This study was conducted in strict accordance with the recommendations contained in the Standard Protocol for Animal Study of Sunchon National University. The protocol was approved by the Sunchon National University Institutional Animal Care and Use Committee (SCNU IACUC; Permission number SCNU IACUC 2016-07). All cells were cultured in a 37°C and 5% CO_2_ incubator. Bone marrow-derived macrophages (BMMs) were derived from unfractionated bone marrow cells (BMCs). The BMCs were isolated from the tibia and femur of 5-week-old male ICR mice (*n* = 2: Damool Science, KR) by flushing with *α*-minimum essential medium (*α*-MEM; Invitrogen Life Technologies, CA, USA) containing 100 U/mL penicillin/streptomycin (Invitrogen, CA, USA). The cells were incubated on a Petri dish in *α*-MEM supplemented with 10% fetal bovine serum (FBS; Invitrogen Life Technologies, CA, USA) and 100 U/mL penicillin/streptomycin (10%  *α*-MEM) with 30 ng/mL of the mouse recombinant macrophage colony-stimulating factor (M-CSF; PEPROTECH, NJ, USA). After 3 days, the cells attached to Petri dishes were obtained as BMMs. The BMMs were plated at a density of 1 × 10^4^ cells/well in a 96-well tissue culture plate in 10%  *α*-MEM and cultured with 10 ng/mL of the mouse recombinant receptor activator of nuclear factor-*κ*B ligand (RANKL; R&D Systems, MN, USA) and 30 ng/mL M-CSF for 4 days in the presence or absence of* C. coreana *Uyeki flos.

### 2.4. Tartrate-Resistant Acid Phosphatase (TRAP) Staining Assay

The cells were washed with PBS and fixed with 3.7% formalin for 5 min. After washing with PBS, the cells were permeabilized with 0.1% Triton X-100 for 10 min. And, then, they were washed, and stained for 10 min at 37°C in the dark with a TRAP solution containing Fast Garnet GBC, sodium nitrite, naphthol AS-BI phosphoric acid, acetate, and tartrate (Sigma-Aldrich, MO, USA). The TRAP+-MNCs (nuclei ≥ 3) were counted as mature osteoclasts.

### 2.5. Cytotoxicity Assay

The BMMs were cultured at a density of 1 × 10^4^ cells/well on a 96-well plate in triplicate with M-CSF (30 ng/mL) and* C. coreana *Uyeki flos. The cell viability was evaluated using a CCK-8 kit (Dojindo Molecular Technologies, Japan) according to the manufacturer's protocol.

### 2.6. Real-Time PCR Analysis

The BMMs were plated at 3.5 × 10^4^ cells/well in a 6-well plate and cultured with 10 ng/mL RANKL and 30 ng/mL M-CSF for 0, 1, 2, and 3 days in the presence or absence of* C. coreana *Uyeki flos. The primers for real-time PCR were designed (Table 1) using the Primer3 design program. The total RNAs were isolated with TRIzol reagent (Invitrogen, CA, USA), and 1 *μ*g of RNAs was reverse-transcribed with the M-MLV cDNA Synthesis kit (Enzynomics, KR) according to the manufacturer's protocol. Quantitative PCR was accomplished using a TOPreal qPCR 2x PreMIX (Enzynomics, KR) and Real-Time PCR detection system (Bio-Rad, CA, USA). All tests were run in triplicate and normalized to the housekeeping gene GAPDH.

### 2.7. Western Blot Analysis

The cells were washed with phosphate-buffer saline (PBS) and homogenized with a lysis buffer containing 20 mM Tris-HCl, pH 7.5, 1% v/v Igepal CA-630, 150 mM NaCl, 1 mM sodium fluoride (Sigma, USA), 1 mM sodium orthovanadate (Sigma, USA), 0.5 mM phenylmethylsulfonyl fluoride (Sigma, USA), 1 mM DTT, 2 mM EDTA, 10 *μ*g/mL aprotinin (Sigma, USA), 5 *μ*g/mL leupeptin (Sigma, USA), and 2 *μ*g/mL pepstatin (Sigma, USA). The lysates were centrifuged at 12,000*g* for 15 min at 4°C. The protein concentration was determined using a DC Protein Assay (Bio-Rad, CA, USA). The protein extracts were subjected to 10% sodium dodecyl sulfate-polyacrylamide gel electrophoresis (SDS-PAGE) (20 *μ*g per lane) and transferred to a polyvinylidene difluoride (PVDF) membrane (Millipore, USA). After blocking with 5% skim milk the membranes were incubated overnight at 4°C with the primary antibodies (NFATc1 and actin antibodies from Santa Cruz Biotechnology, CA, USA). After washing, the membranes were incubated with the horseradish peroxidase- (HRP-) conjugated secondary antibody (Santa Cruz Biotechnology, CA, USA) for 2 hrs at room temperature. The antibody blots were developed using a MicroChemi 4.2 (DNR Bio-Imaging System, Jerusalem, Israel) and Super-Signal West Pico/Femto Chemiluminescent Substrate (Pierce Chemical, IL, USA).

### 2.8. Resorption Pit Analysis

To analyze the surface for pit formation, the BMMs were plated at 6 × 10^6^ cells/well in a 24-well tissue culture plate and cultured with the conditions of 30 ng/mL M-CSF in a negative/positive (without/with 10 ng/mL RANKL) and the presence (10 and 30 *μ*g/mL) of 80%* C. coreana *Uyeki flos for 4 days. The media were discarded and 100 *μ*L of 10% bleach solution was changed on day 4. And cells were incubated for 5 min at RT. The plate was washed twice with distilled water and dried at RT for about 4 hrs. The generated pits were observed using a microscope at 100x magnification.

### 2.9. Statistical Analysis

The proteins and RNA from* C. coreana *Uyeki flos and control groups were analyzed to determine the differences in the levels of expression using Student's *t*-test. Probability (*P*) values less than 0.05 were considered significant (^*∗*^ *P* < 0.05, ^*∗∗*^ *P* < 0.01, and ^*∗∗∗*^ *P* < 0.001).

## 3. Results

### 3.1. *C. coreana* Uyeki Flos Inhibits the Differentiation of BMMs into Osteoclasts

**Figure 1:**
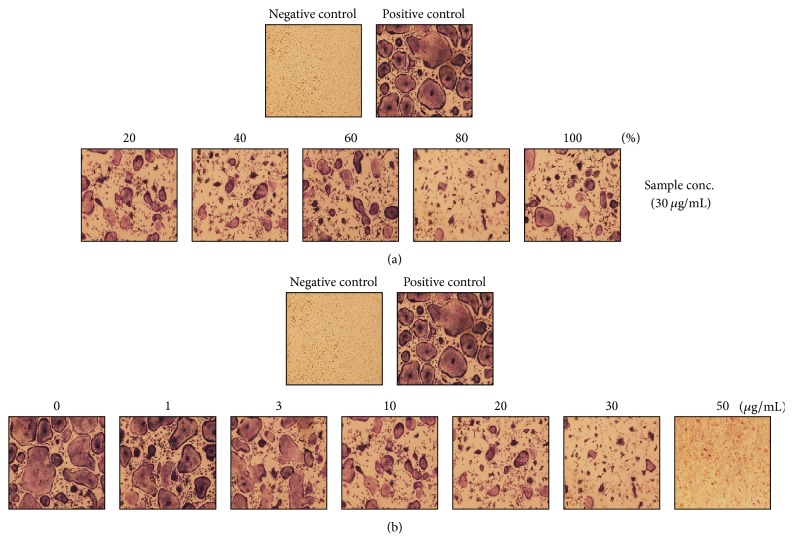
*Osteoclast differentiation was inhibited by C. coreana Uyeki flos extracts.* BMMs were cultured for 4 days in 0.1% DMSO (control vehicle) or the concentration 30 *μ*g/ml of* C. coreana *Uyeki flos extract with RANKL (10 ng/mL) and M-CSF (30 ng/ml). The inhibitive patterns of osteoclast differentiation based on the alcoholic percentage of extracts (a). The inhibitive patterns of osteoclast differentiation based on the concentration of the 80% alcoholic extract of* C. coreana *Uyeki flos (b).

**Figure 2:**
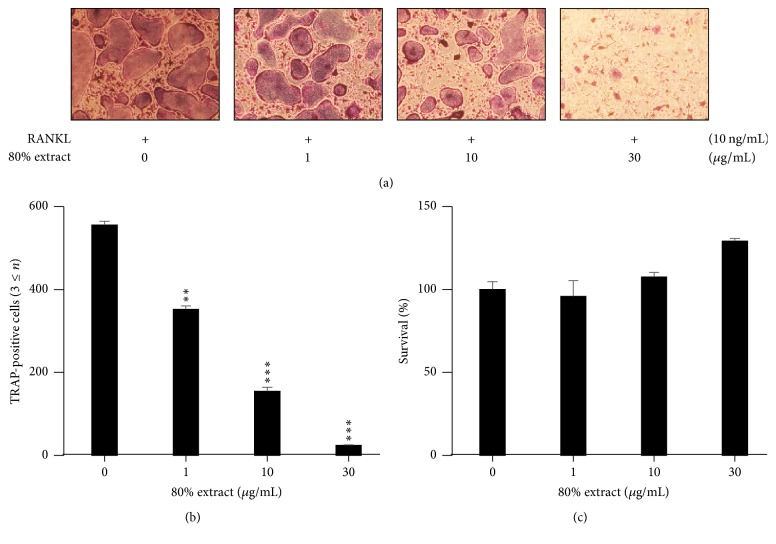
*RANKL-mediated osteoclastogenesis was suppressed by C. coreana Uyeki flos extracts, and the extracts had no cytotoxic effect.* (a) BMMs were cultured for 4 days in 0.1% DMSO (control vehicle) or the indicated concentrations of* C. coreana *Uyeki flos extract with RANKL (10 ng/mL) and M-CSF (30 ng/ml). Multinucleated cells were fixed (3.7% formalin), permeabilized (0.1% Triton X-100), and stained with the TRAP solution. (b) Mature TRAP-positive multinucleated osteoclasts (MNCs) were photographed under an optical microscope. TRAP-positive MNCs (nuclear number > 3) were counted. ^*∗∗*^ *P* < 0.01; ^*∗∗∗*^ *P* < 0.001. (c) The effect of* C. coreana *Uyeki flos extracts on the viability of BMMs was measured by the CCK-8 assay.

To examine the effects of* C. coreana* Uyeki flos on RANKL-induced osteoclast differentiation, we performed* in vitro* osteoclast differentiation. The BMMs were treated with the vehicle (0.1% DMSO) as a control and the* C. coreana* Uyeki flos extract and then mixed with 30 ng/mL M-CSF and 10 ng/mL RANKL for 4 days. We conducted the TRAP staining to determine the most effective alcohol concentrations; the inhibitory effect of osteoclastogenesis was highest with 80% alcohol extract (Figure 1(a)). We performed the experiment on the optimal amounts of samples to inhibit osteoclast differentiation. The 80% extract of* C. coreana* Uyeki flos at a concentration above 10 *μ*g/mL inhibited the osteoclast differentiation. Its inhibitory effect appeared as the plateau above 30 *μ*g/mL (Figures 1(b) and 2(a)). Moreover, the 80% extract of* C. coreana* Uyeki flos at a concentration above 10 *μ*g/mL inhibited the differentiation of TRAP^+^-osteoclasts. The number of TRAP^+^-MNCs (nuclei ≥ 3) was decreased dose-dependently by* C. coreana* Uyeki flos (Figure 2(b)).

### 3.2. *C. coreana* Uyeki Flos Had No Cytotoxic Effect

The toxic effects of* C. coreana* Uyeki flos on the BMMs were identified. The BMMs were incubated with* C. coreana* Uyeki flos-treated medium containing 30 ng/mL M-CSF for 3 days and the cell viability was measured by the CCK-8 kit (Figure 2(c)).* C. coreana* Uyeki flos at concentrations less than 30 *μ*g/mL showed no cytotoxicity to the BMMs.

### 3.3. Main Components Identified from the 80% Alcoholic Extracts

Table 2 listed total flavonoids, phenolics, and the contents of active constituents of phytochemicals. The 80% alcoholic extract of* C. coreana* Uyeki flos was composed of 269 mg of total phenolics, 16 mg of total flavonoids, and some phytochemicals, such as bergenin (BER), quercetin (QCT), and quercitrin (QCIT). The contents of BER, QCT, and QCIT were 17.01, 1.50, and 0.05% (w/w), respectively, in the 80% alcoholic extract.

### 3.4. *C. coreana* Uyeki Flos Suppresses RANKL-Induced Gene Expression

**Figure 3:**
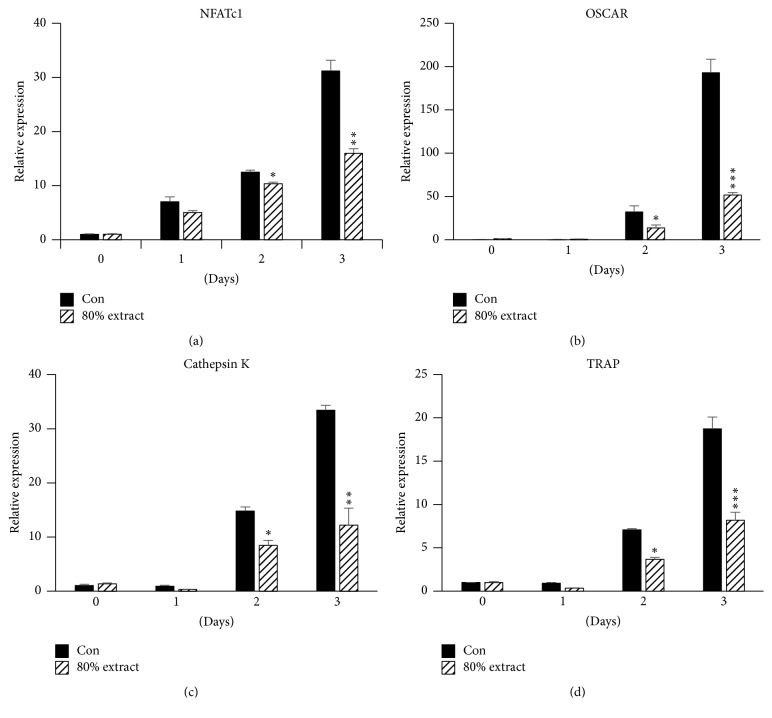
*Effect of C. coreana Uyeki flos extracts on RANKL induced the mRNA expressions of osteoclastic-specific genes.* The BMMs were pretreated with vehicle (0.1% DMSO) or* C. coreana *Uyeki flos extracts (30 *μ*g/mL) for 1 hr and then treated with RANKL (10 ng/mL) and M-CSF (30 ng/mL) for the designated time periods. The total RNA was extracted using TRIzol reagent, and mRNA expression levels of NFATc1, OSCAR, Cathepsin K, and TRAP were measured by real-time PCR. The internal control was used with GAPDH. ^*∗*^ *P* < 0.05; ^*∗∗*^ *P* < 0.01; ^*∗∗∗*^ *P* < 0.001.

Real-time PCR analysis was performed to confirm the effects of* C. coreana* Uyeki flos on osteoclast formation and the expression of transcription factors and osteoclast-specific markers.* C. coreana* Uyeki flos reacted with RANKL and inhibited the mRNA expression of OSCAR and NFATc1, which are important transcription factors involved in osteoclast differentiation (Figures 3(a) and 3(b)). In addition, the mRNA expression levels of cathepsin K and TRAP involved in NFATc1 expression were inhibited significantly in osteoclast differentiation (Figures 3(c) and 3(d)).

### 3.5. *C. coreana* Uyeki Flos Suppresses RANKL-Induced Protein Expression of NFATc1

**Figure 4:**
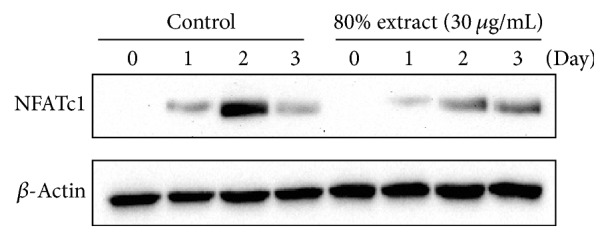
*Effect of C. coreana Uyeki flos extracts on the translational expression of an osteoclast-specific transcription factor, NFATc1.* BMMs were pretreated with vehicle (0.1% DMSO) or* C. coreana *Uyeki flos extracts (30 *μ*g/mL) for 1 hr prior to RANKL (10 ng/mL) and M-CSF (30 ng/mL) treatment at the designated time periods. *β*-Actin was used as the internal control.

The effect of* C. coreana* Uyeki flos on the protein level of NFATc1, a key regulator of osteoclast differentiation, was determined by western blotting. RANKL increased the protein expression of NFATc1 dramatically over time, but the level of NFATc1 protein expression was reduced significantly after exposure to* C. coreana* Uyeki flos (Figure 4). This result suggested that* C. coreana* Uyeki flos inhibited the protein expression of NFATc1, which is key element in the osteoclast formation process.

### 3.6. *C. coreana* Uyeki Flos Suppresses RANKL-Induced Resorptive Activity

**Figure 5:**
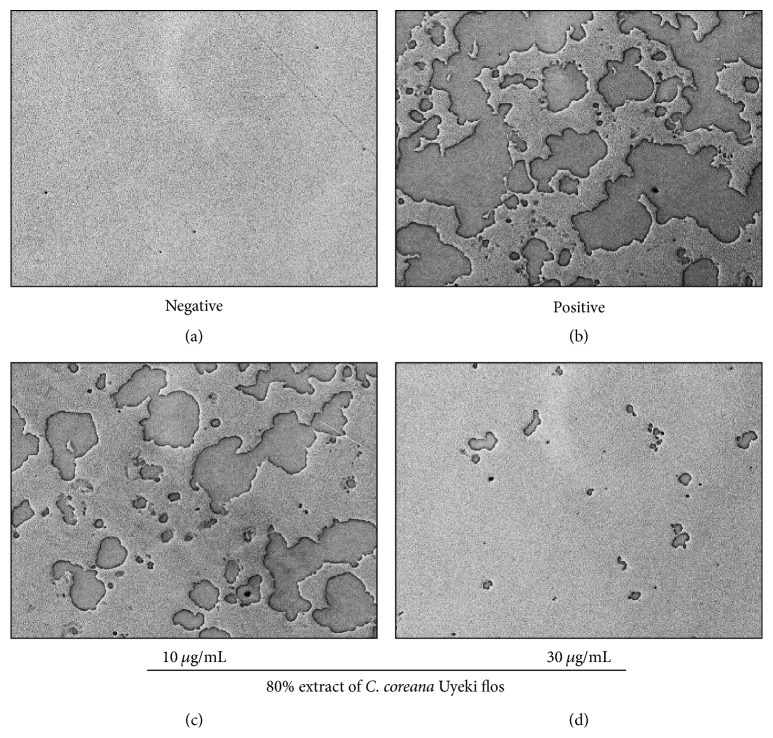
*Resorption pit assay for osteoclast.* The BMMs were plated at 6 × 10^6^ cells/well in a 24-well tissue culture plate and cultured with the conditions of 30 ng/mL M-CSF in a negative/positive (without/with 10 ng/mL RANKL) ((a) and (b), resp.) and the presence (10 and 30 *μ*g/mL, (c) and (d), resp.) of 80%* C. coreana *Uyeki flos for 4 days.

To confirm the inhibitory activity of the alcoholic extract of* C. coreana *Uyeki flos in osteoclast differentiation, we performed the resorption pit assay that can resorb the mineralization matrix and indicate the ability to form resorption pits on the surface. This experiment showed a lot of pits produced by human osteoclast precursor cells on the Corning Osteo Assay Surface in a positive well, compared with a negative well (Figures 5(a) and 5(b)). It was confirmed that the Corning Osteo Assay Surface was a good substrate for the analysis of osteoclast functional activity. When the alcoholic extract of* C. coreana *Uyeki flos was added, the number of resorptive pits was decreased in proportion to the adding amounts of the extract (Figures 5(c) and 5(d)). This result demonstrated that the alcoholic extract of* C. coreana *Uyeki flos inhibited the resorptive activity of osteoclast induced by RANKL.

## 4. Discussion

Imbalances between osteoblasts and osteoclasts induced by various factors cause excessive bone resorption, which is particularly characteristic of osteoporosis. In the bone marrow, osteoclastic bone cells differentiate from hematopoietic stem cells [9, 10]. Osteoclastic cells are often differentiated* in vitro* when BMMs are treated with RANKL and M-CSF [11]. Activation of the RANKL-induced NF-kB and MAPK pathways is an important mechanism for osteoclastogenesis [12–14]. In this study, the alcoholic extracts derived from* C. coreana* Uyeki flos inhibited osteoclast differentiation from the BMMs and downregulated the expression of the marker genes and involved protein by blocking the RANKL-induced signaling pathway [15]. In addition, we confirmed that the osteoclast suppressed by* C. coreana* Uyeki flos had a resorptive activity using the resorption pit assay. These results suggested that* C. coreana* Uyeki flos could be used as a therapeutic agent to inhibit bone breakdown in osteolytic bone diseases such as osteoporosis.

A number of flavonoids and phenolic compounds possess antiosteoclastic activity [16]. Therefore, the ethanol extraction process was optimized with respect to the total flavonoids and total phenolics. In a previous study, the 80% alcoholic extract showed the optimal flavonoid and phenolic content [7, 8]. Therefore, according to the results of a previous study and the findings of the current study, the 80% alcoholic extract was selected for further* in vitro* studies. A previous study showed that BER, QCT, and QCIT were the main bioactive phytochemicals identified from* C. coreana* Uyeki flos [7]. The 80% alcoholic extract exhibited the highest antiosteoclastic activity among all extracts examined (Figures 1 and 2). Therefore, these constituents were analyzed in the 80% alcoholic extract prepared in the present study. BER was one of the major compounds in the 80% alcoholic extract of* C. coreana *Uyeki flos. Nazir et al. reported that BER inhibits the formation of proinflammatory helper T cell type 1 cytokines (TNF-*α*, IFN-*γ*, and IL-2), while it potentiates the anti-inflammatory helper T cell type 2 cytokines (IL-5 and IL-4) in the arthritic mice model [17]. QCT and QCIT appear to be important active compounds in the 80% alcoholic extract. Oliveira et al. reported that QCT did not affect the cell viability and decreased osteoclast formation compared with a control and downregulated NF-*κ*B pathway. They suggested that QCT might be inhibitor of osteoclast differentiation under inflammatory conditions (LPS-induced) through attenuation of NF-*κ*B activation [18]. Previous studies have reported that QCT increased bone formation marker, such as osteocalcin. Derakhshanian et al. suggested that quercetin prevented the glucocorticoid-induced osteoporosis by its bone enhancing effect [19]. Therefore, QCT is a potential substance in the extract of* C. coreana* that induces the activity of antiosteoclast and proosteoblast. The antiosteoclastic activity of QCT by osteoclast differentiation* in vitro* assay in our lab (data not shown) was confirmed. Satué et al. reported that QCIT promotes osteoblastogenesis in MC3T3-E1 cells and inhibits osteoclast differentiation in RAW264.7 cells, highlighting the positive effect of QCIT on the prevention of osteoporosis [20].

Several proteins are involved in the functioning of osteoclast differentiation. Some of the important proteins were analyzed by real-time qPCR. The OSCAR presents a costimulatory receptor that is important for osteoclast differentiation and is involved in the activation of NFATc1 and macrophage immune cell regulators [21]. Therefore, OSCAR is associated with osteoporosis and rheumatoid arthritis and may trigger their onset mechanism [22, 23]. Cathepsin K is expressed mainly in osteoclasts and acts as a lysosomal cysteine protease [24]. The protein also plays a significant role in degrading the organic composition of bone (predominantly Type I collagen) [25]. Cathepsin K plays a significant role in bone resorptive mechanism. Odanacatib (ODN), an inhibitor of cathepsin K, is currently being developed as a treatment for osteoporosis [26]. TRAP is expressed strongly in osteoclastic cell and induces osteoclast differentiation [27, 28]. TRAP is involved in osteoclast migration to the spot of bone resorption and begins osteoclast differentiation [29]. In this study, TRAP, OSCAR, and cathepsin K, which are important mediators of osteoclast differentiation, were inhibited by the treatment with* C. coreana* Uyeki flos.

Interestingly, unlike the expression pattern of increasing expression of NFATc1 mRNA in the presence of RANKL until day 3, the level of translational expression of NFATc1 peaked at day 2 and rapidly decreased to baseline by day 3. Because of the nuclear import of these proteins in the presence of RANKL, the expression level of NFATc1 protein was decreased during late-stage osteoclast differentiation by an auto-feedback system of NFATc1 but the mRNA expression level of NFATc1 is maintained until the last stage of osteoclastogenesis [30].

## 5. Conclusions

In the present study, the alcoholic extracts of* C. coreana* Uyeki flos were prepared, and the ethanol extraction process was optimized with respect to the total flavonoid, total phenolic content, contents of active compounds, and antiosteoclastic activity. The* in vitro *study showed that the optimized 80% alcoholic extract could inhibit osteoclast differentiation significantly at a concentration of 30 *μ*g/mL. In addition, the osteoclast inhibited by* C. coreana* Uyeki flos had a resorptive activity; that is, the resorptive activity was suppressed by the extract of* C. coreana* Uyeki flos. The extract of* C. coreana* Uyeki flos was analyzed by HPLC and its contents were identified.* In vitro* study showed that the antiosteoclastic activity of the extract was caused by total phenolics, flavonoids, and some phytochemicals, such as QCT and QCIT, synergistically. This paper is the first scientific report of the antiosteoclastic activity of* C. coreana *Uyeki flos extracts, highlighting the feasibility of the present herbal medicine for the treatment of osteoporosis.

## Figures and Tables

**Table 1:** Primer sequences used in this study.

Gene of interest	Direction	Primer sequence (5′–3′)
TRAP	Sense	GATGACTTTGCCAGTCAGCA
	Antisense	ACATAGCCCACACCGTTCTC
NFATc1	Sense	GGGTCAGTGTGACCGAAGAT
	Antisense	GGAAGTCAGAAGTGGGTGGA
Cathepsin K	Sense	GGCCAACTCAAGAAGAAAAC
	Antisense	GTGCTTGCTTCCCTTCTGG
OSCAR	Sense	CTGCTGGTAACGGATCAGCTC
	Antisense	CCAAGGAGCCAGAACCTT
GAPDH	Sense	AACTTTGGCATTGTGGAAGG
	Antisense	ACACATTGGGGGTAGGAACA

**Table 2:** Total flavonoids, phenolics, and contents of active constituents of the main phytochemicals identified from the 80% ethanolic extract of *C. coreana* Uyeki flos (*n* = 5).

Constituent	Content
Total flavonoids	16.43 ± 0.09 (mg/g eq.quercetin)
Total phenolics	269.37 ± 98.72 (mg/g eq.gallic acid)
Bergenin	17.01 ± 0.08% (w/w)
Quercetin	1.5 ± 0.007% (w/w)
Quercitrin	0.05 ± 0.002% (w/w)
